# Calycosin (CA) inhibits proliferation, migration and invasion by suppression of CXCL10 signaling pathway in glioma

**DOI:** 10.18632/aging.205572

**Published:** 2024-03-09

**Authors:** Xiaoyu Zheng, Danmin Chen, Menghui Li, Jianchen Liao, Liqun He, Lu Chen, Rong Xu, Maoying Zhang

**Affiliations:** 1 Department of Intensive-Care Unit, Affiliated First Hospital, Jinan University, Guangzhou 510630, China; 2 Department of Neurosurgery, Affiliated Shunde Hospital, Jinan University, Shunde, Foshan 528000, China; 3 Department of Neurosurgery, The Second Affiliated Hospital of Guangzhou Medical University, Guangzhou 510260, China; 4 Department of Neurosurgery, Affiliated First Hospital, Jinan University, Guangzhou 510630, China; 5 Department of Operating Room, Guangzhou Tianhe Longdong Hospital, Guangzhou 510520, China

**Keywords:** calycosin (CA), CXCL10, glioblastoma, inflammation

## Abstract

Glioblastoma is the most common malignant tumor in the central nervous system and its occurrence and development is involved in various molecular abnormalities. C-X-C chemokine ligand 10 (CXCL10), an inflammatory chemokine, has been reported to be related to the pathogenesis of cancer while it has not yet been linked to glioma. Calycosin, a bioactive compound derived from Radix astragali, has demonstrated anticancer properties in several malignancies, including glioma. Nonetheless, its underlying mechanisms are not fully understood. This study explores CXCL10 as a potential therapeutic target for calycosin in the suppression of glioblastoma. We observed that CXCL10 expression correlates positively with glioma malignancy and inversely with patient prognosis, highlighting its potential as a glioblastoma treatment target. Furthermore, we found that calycosin inhibited proliferation, migration, and invasion in U87 and U251 glioma cells, and decreased CXCL10 expression in a dose-dependent manner, along with its downstream effectors such as NLRP3, NF-κB, and IL-1β. Additionally, molecular docking experiments demonstrated that calycosin exhibits a notable binding affinity to CXCL10. Overexpression of CXCL10 counteracted the inhibitory effects of calycosin on cell proliferation, migration, and invasion, while CXCL10 knockdown enhanced these effects. Finally, we verified that calycosin inhibited glioma growth in a xenograft mouse model and downregulated CXCL10 and its downstream molecules. These findings suggest that targeting CXCL10 may be an effective strategy in glioblastoma treatment, and calycosin emerges as a potential therapeutic agent.

## INTRODUCTION

Glioblastoma (GBM) is the most common and aggressive malignant brain tumor in the central nervous system. GBM treatment typically involves surgery, complemented by chemotherapy and radiotherapy [1, 2]. However, due to its multifocal nature and high invasiveness, complete surgical resection is often challenging, leading to a high recurrence rate and a median survival of only 14.6 months post-diagnosis [3]. Consequently, developing novel and effective therapeutic methods for GBM is imperative.

Calycosin (CA), a major bioactive compound extracted from Radix astragali, is an isoflavonoid and phytoestrogen [4]. It has demonstrated various pharmacological properties, including anti-inflammatory, neuroprotective, and cardiovascular effects [5–7]. Recent studies have highlighted its anti-tumor activities; for instance, calycosin suppresses breast cancer via the downregulation of the Foxp3/VEGF/MMP-9 signaling pathway [8]. Additionally, it induces apoptosis in osteosarcoma through the ERβ-mediated PI3K/Akt signaling pathways [9]. Notably, Nie et al. reported calycosin’s anti-GBM effects through TGFβ inhibition [10, 11]. However, the underlying mechanisms remain to be fully elucidated.

Chemokines, small secretory immunoregulatory proteins, regulate cell trafficking through interactions with seven-transmembrane G protein-coupled receptors (GPCRs) and are implicated in oncogenesis, including tumor development and metastatic spread. CXCL10, also known as interferon-gamma-induced protein 10 (IP-10), is a 10 kDa polypeptide of the CXC chemokine subfamily. It plays multiple roles, such as mediating immune cell chemotaxis, angiogenesis, and inflammation response [12–14]. Recent studies have linked CXCL10 to the progression of various cancers, including pancreatic, breast, ovarian, and colorectal cancer [15–18]. However, its role as either a tumor suppressor or contributor in glioma remains controversial.

Inflammation significantly promotes tumorigenesis onset and progression [19], as observed in GBM, where it facilitates cancer progression and treatment resistance [20]. The NOD-like receptor protein-3 (NLRP3) inflammasome, excessively activated in various cancers including glioma, and its suppression has been shown to reduce tumor growth and prolong survival in glioma-bearing mice. The NLRP3 downstream effectors, IL-1β and NF-κB, are abundantly present in the tumor microenvironment of glioblastomas, contributing to their development [21–23]. However, the relationship between CXCL10 and NLRP3 is not well understood, and whether CA can modulate CXCL10 to inhibit GBM remains unknown.

In this study, we demonstrated that high CXCL10 expression is associated with higher pathological grades and poorer prognosis. We also identified CXCL10 as a potential therapeutic target for CA in GBM suppression, via downregulating downstream molecules such as NLRP3, NF-κB, and IL-1β, both *in vivo* and *in vitro.* These findings suggest that CXCL10 is a novel biomarker in glioblastoma, and calycosin may serve as a potential therapeutic agent.

## RESULTS

### CXCL10 was upregulated in glioma tissues and contributes to poor prognosis

**Figure 1:**
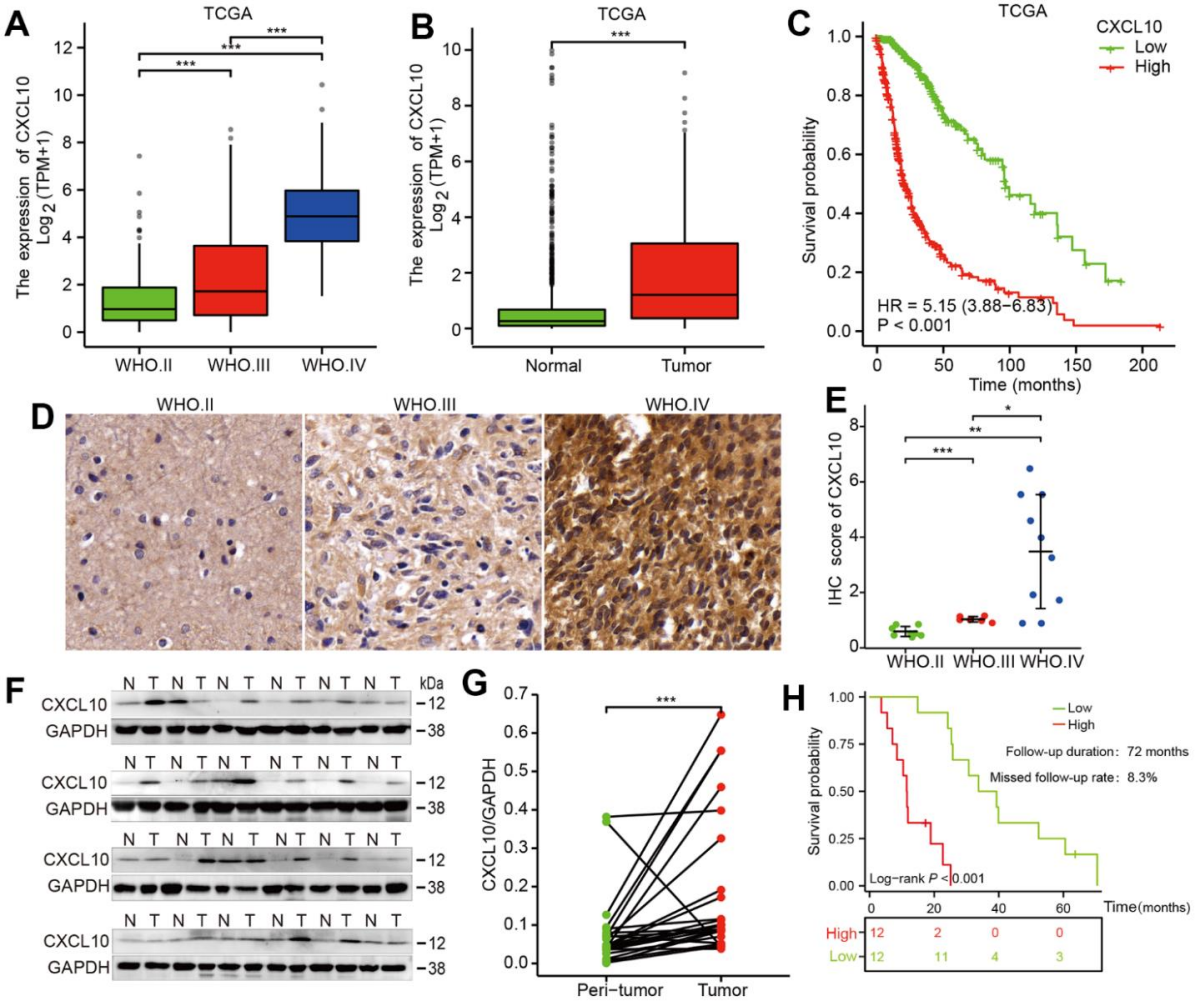
**CXCL10 is upregulated in glioma tissues and positively associated with tumor progression and poor prognosis.** (**A**) Statistical evaluation of CXCL10 expression across different grades of glioma in the TCGA database. (**B**) analysis of CXCL10 expression in GBM tumor tissues compared to normal brain tissues using data from the TCGA database. (**C**) Survival curves showing the overall survival (OS) stratified by CXCL10 expression levels in glioma patients, based on TCGA data. (**D**, **E**) Comparative analysis of CXCL10 expression in 24 paired tumor and peritumor tissues from glioma patients. (**F**, **G**) examination of CXCL10 expression in GBM tumor tissues compared to normal brain tissues in 24 glioma cases. (**H**) OS analysis in a cohort of 24 glioma patients stratified by CXCL10 expression.

Our study evaluated the mRNA expression of CXCL10 using public data from The Cancer Genome Atlas (TCGA) and the GTEx database, as well as 24 pairs of glioma tissue samples. Analysis of the TCGA database revealed a significant increase in CXCL10 mRNA expression correlating with higher WHO glioma grades (Figure 1A). Additionally, CXCL10 levels were notably higher in glioma tissues compared to normal tissues (Figure 1B). High CXCL10 expression was associated with markedly poorer overall survival (OS) in patients (Figure 1C). Similarly, in our 24 paired human glioma samples, CXCL10 expression increased with glioma grades (Figure 1D, 1E and Supplementary Table 1) and was more pronounced in glioma than in adjacent normal brain tissues (Figure 1F, 1G). Patients with high CXCL10 expression also exhibited shorter survival periods (Figure 1H). These findings suggest a positive correlation between high CXCL10 expression and poor prognosis in glioma patients.

### CA inhibited cells growth and downregulated CXCL10 signaling in GBM cells

**Figure 2:**
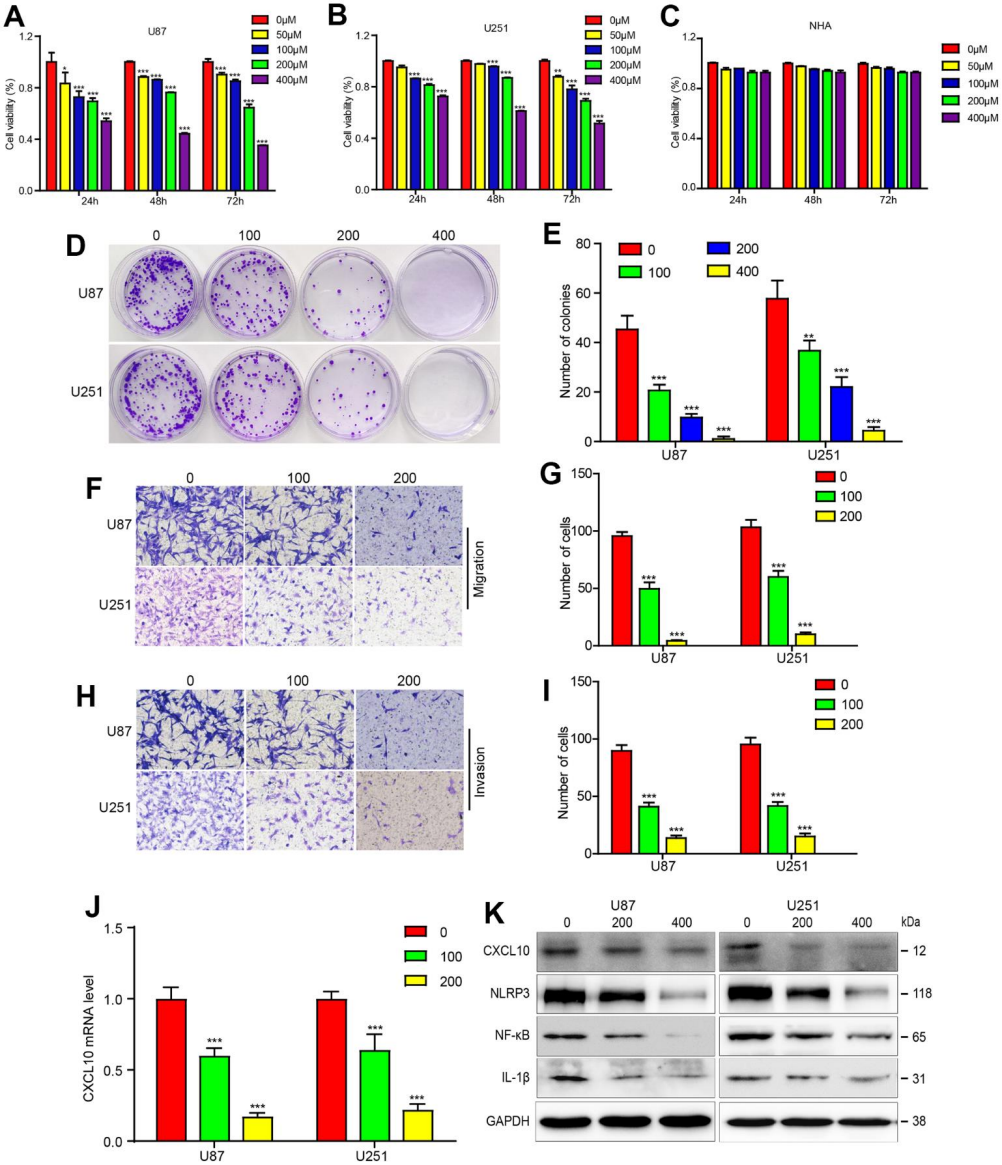
**Calycosin suppressed GBM progression and downregulated CXCL10 pathway in U87 and U251 cells.** (**A**–**C**) The CCK8 experiment were performed in U87, U251 and HNA cells with the different concentrations of calycosin. (**D**, **E**) Cell colony formation in glioblastoma cells with CA treatments as indicated dose. (**F**–**I**) U87 and U251 cells were treated with the indicated dose of CA for 24 hours, and transwell assay was applied to examine the migration and invasion. (**J**) RT-PCR was applied to detect the mRNA level after indicated dose of CA. (**K**) U87 and U251 cells were performed with the different dose of CA for 24 hours and western bolt was applied to detect the protein expression levels of CXCL10 pathway. *P<0.05, **P<0.01, ***P<0.001, compared with control (0μM).

To evaluate CA’s potential effects on GBM, CCK-8 assays were conducted. Results showed that CA treatment significantly inhibited cell proliferation in U87 and U251 GBM cells in a dose- and time-dependent manner, with cell viability decreasing from 90% to 40% as CA concentration increased from 100 to 400 μM. This effect was not observed in HNA (Human Normal Astrocytes) (Figure 2A–2C). Colony formation assays further confirmed CA’s inhibitory effect on U87 and U251 cell growth (Figure 2D, 2E). Cell migration and invasion assays demonstrated that calycosin treatment significantly reduced these activities in GBM cells in a dose-dependent manner (Figure 2F–2I). These results indicate that calycosin effectively inhibits GBM cell migration and invasion.

As CXCL10 is upregulated in GBM tissues and contributes to tumor progression and poor prognosis, inhibiting CXCL10 could be a viable treatment approach. Treatment with varying concentrations of CA downregulated CXCL10 expression at both mRNA and protein levels, reducing downstream molecules such as NLRP3, NF-κB, and IL-1β (Figure 2J, 2K).

### Overexpression of CXCL10 reduced the effects of calycosin in GBM cells

**Figure 3:**
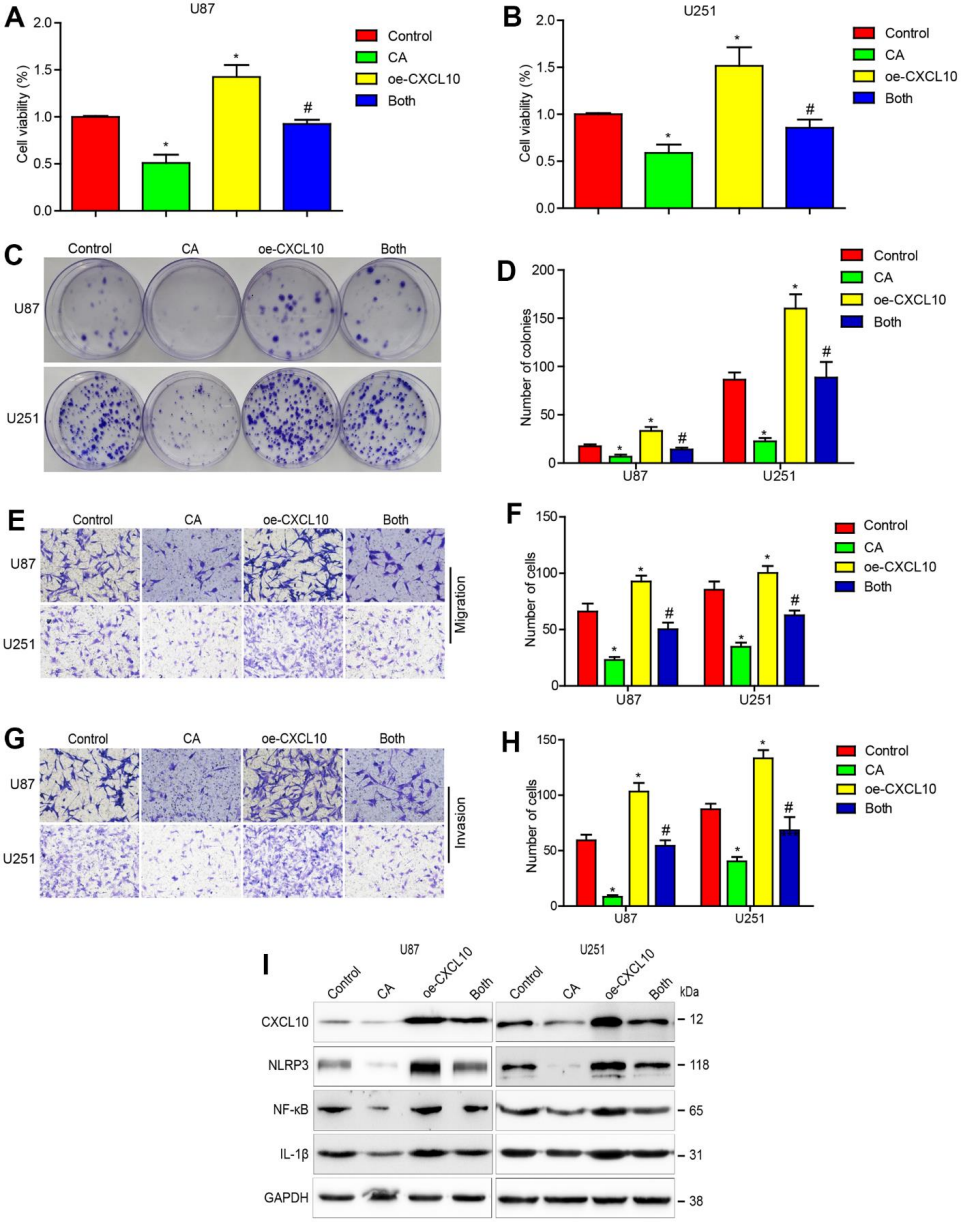
**CXCL10 overexpression reduces the effects of calycosin on cells proliferation, migration, invasion and CXCL10 signaling in GBM.** (**A**–**D**) Effect of CXCL10 overexpression on calycosin in U87 and U251 cell proliferation. (**E**–**H**) Effect of CXCL10 overexpression on calycosin in U87 and U251 cell migration and invasion. (**I**) Overexpressing CXCL10 rescues calycosin-induced CXCL10, NLRP3, NF-κB and IL-1β downregulation. Control: GFP lentivirus transfection; CA: GFP lentivirus transfection + 200μM calycosin; oe-CXCL10: lentivirus transfection CXCL10; Both: lentivirus transfection CXCL10 + 200μM calycosin. *P < 0.05 vs control. ^#^P < 0.05, compared with either CA treatment or CXCL10 transfection alone.

To determine if calycosin’s suppression of GBM cells is mediated through downregulating CXCL10, U87 and U251 cells were infected with a lentiviral vector carrying CXCL10 cDNA. Overexpression of CXCL10 enhanced cell proliferation, migration, and invasion, and reduced the inhibitory effects of CA (Figure 3A–3H). Overexpressed CXCL10 also promoted NLRP3, NF-κB, and IL-1β expression, countering the suppressive effects of calycosin treatment (Figure 3I).

### Down-regulation of CXCL10 enhanced the effects of calycosin in GBM cells

**Figure 4:**
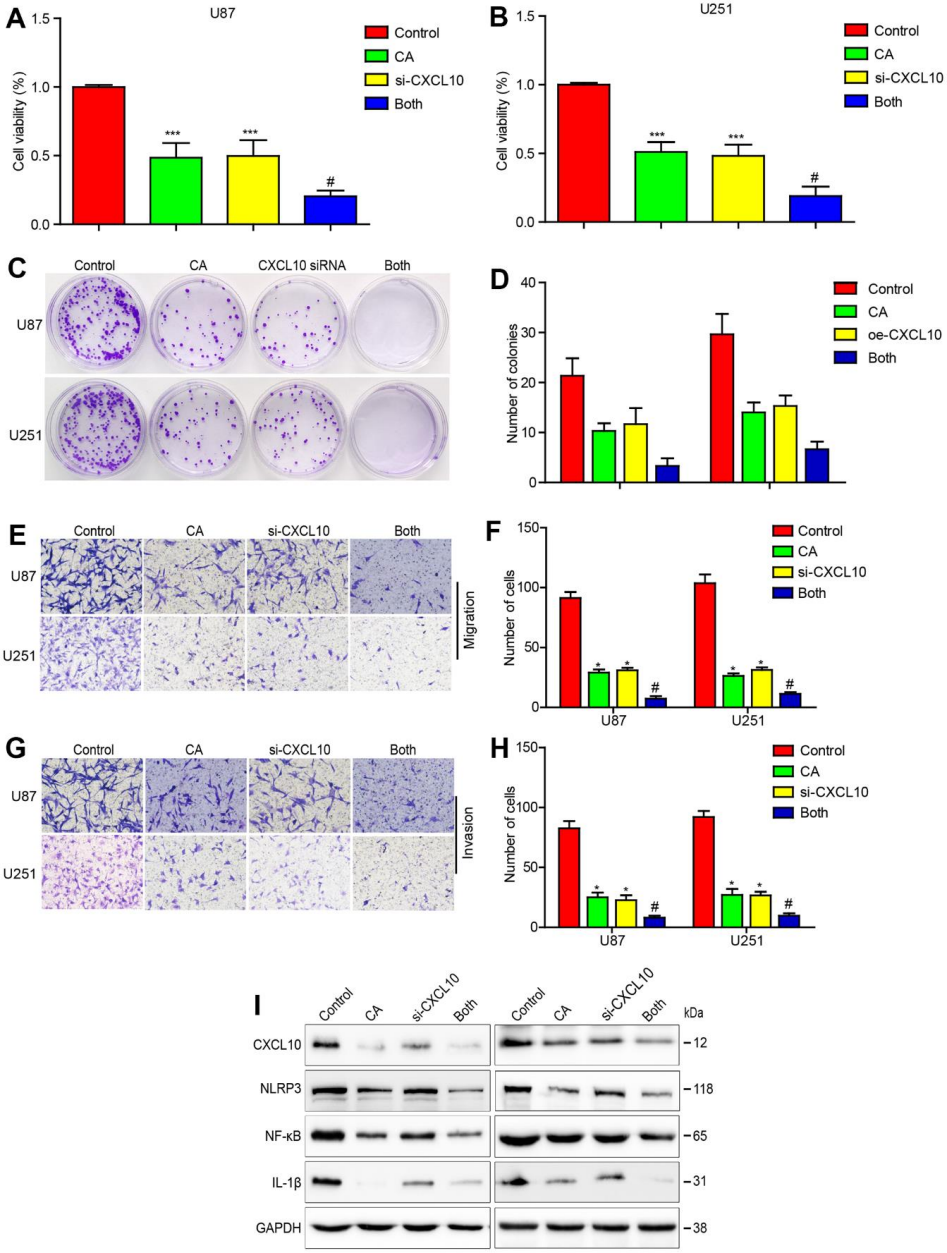
**Downregulating CXCL10 enhances the effects of calycosin on cell proliferation, migration, invasion and CXCL10 signaling in GBM.** (**A**–**D**) Effects of CXCL10 knockdown on calycosin in U87 and U251 cell proliferation. (**E**–**H**) Effects of downregulating CXCL10 on calycosin in U87 and U251 cell migration and invasion. (**I**) Downregulating CXCL10 promotes calycosin-induced CXCL10, NLRP3, NF-κB and IL-1β downregulation. Control: siRNA negative control transfection; CA: siRNA negative control transfection + 200μM calycosin; CXCL10: CXCL10 siRNA transfection; both: CXCL10 siRNA transfection + 200μM calycosin. *P < 0.05 vs control. ^#^P < 0.05, compared with either CA treatment or CXCL10 siRNA alone.

To further confirm CXCL10’s oncogenic role in CA-mediated anticancer effects, CXCL10 was knocked down in glioblastoma cells using specific siRNA. CCK-8 and colony formation assays showed enhanced suppression of cell proliferation following CXCL10 knockdown in calycosin-treated cells (Figure 4A–4D). Cells treated with CXCL10 siRNA or calycosin exhibited potent inhibition of migration and invasion (Figure 4E–4H). This combination treatment showed greater efficacy than either CXCL10 siRNA or calycosin alone. Calycosin treatment also decreased the expression of NLRP3, NF-κB, and IL-1β, with further suppression observed upon CXCL10 downregulation (Figure 4I). These results confirm calycosin’s anticancer function, partly through downregulating CXCL10.

### Calycosin inhibited GBM growth in a U87 xenograft mouse model

**Figure 5:**
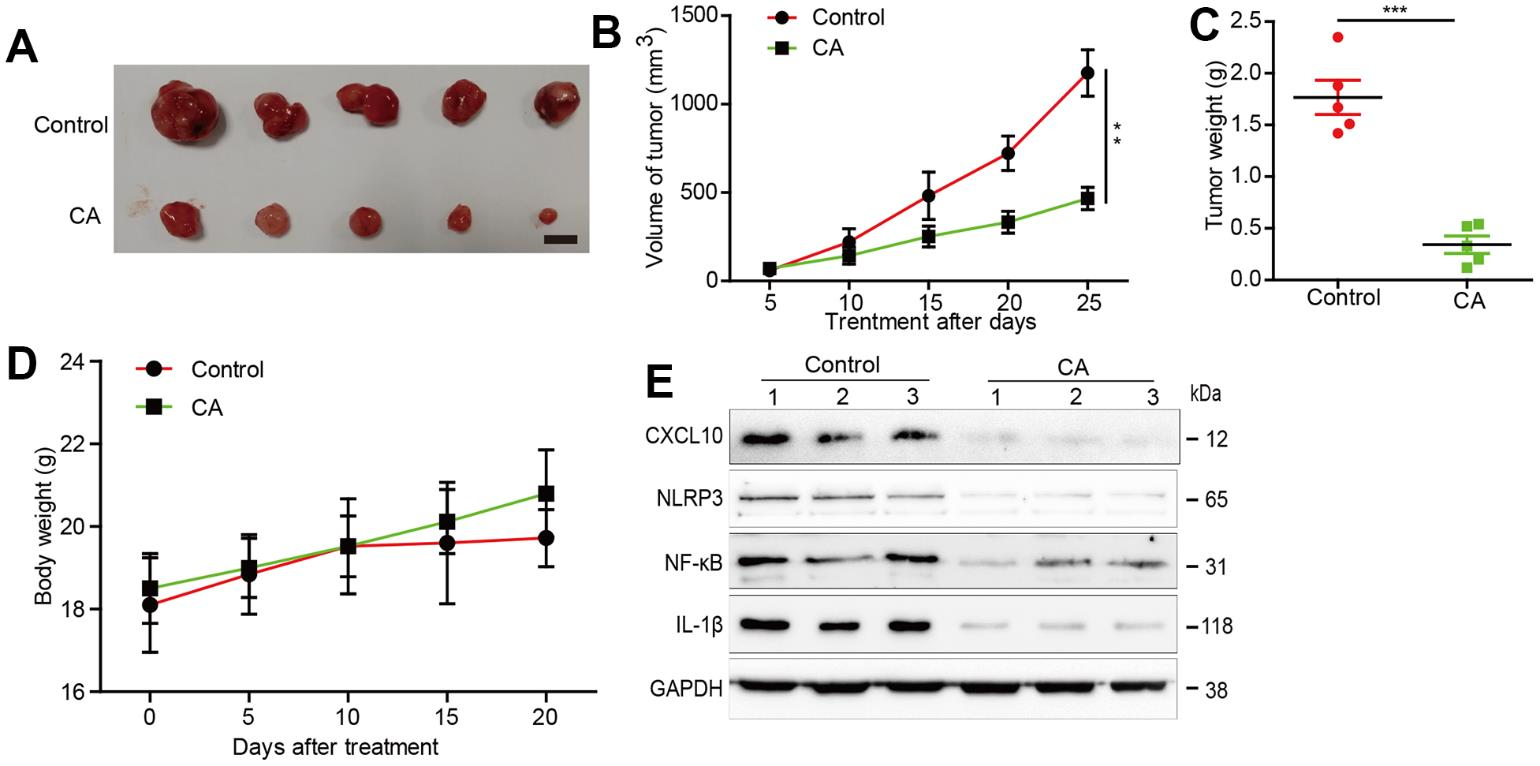
**Effects of calycosin in xenograft mouse models.** (**A**) Inhibition in the size of the xenograft U87 tumors were photographed. (**B**) The tumor volume and body weight were measured per 5 days. (**C**) At the end of the experiments, tumor was excised from the mice and the weigh is measured. (**D**) The mice’s body weight was measure at the indicated time point. (**E**) At the end of the experiment or after the mice dead, tumor tissues were excised from the mice and the protein lysates were performed to estimated protein expression *P<0.05, compared with control. Scale =1cm.

The inhibitory effects of calycosin on glioblastoma progression were further explored in a U87 xenograft mouse model. Calycosin-treated mice exhibited significantly smaller tumor volumes compared to the vehicle-treated group, without changes in body weight (Figure 5A–4D). CXCL10 expression and its pathway components, NLRP3, NF-κB, and IL-1β, were markedly suppressed in tumor tissues from the calycosin-treated mice (Figure 5E). These *in vivo* results corroborate our *in vitro* findings, demonstrating calycosin’s critical role in suppressing glioblastoma growth through CXCL10 inhibition.

### Calycosin’s interaction with CXCL10

**Figure 6:**
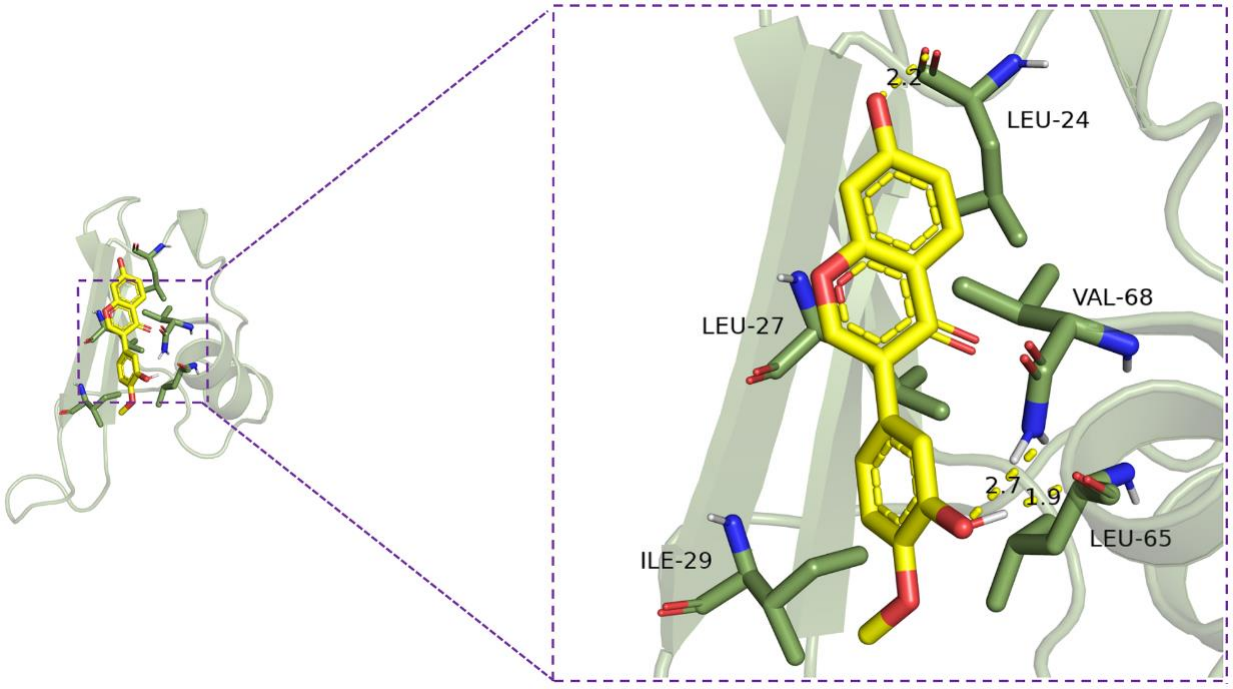
Calycosin’s interaction with CXCL10.

In order to evaluate the potential interaction between calycosin and CXCL10, we conducted molecular docking experiments. These experiments demonstrated that calycosin exhibits a notable binding affinity to CXCL10, characterized by a binding energy of -6.8 kcal/mol. The interaction is primarily mediated through the formation of hydrogen bonds with amino acids LEU-65, VAL-68, and LEU-24 (Figure 6). These findings indicate a stable binding conformation and suggest that calycosin may have an inhibitory effect on CXCL10 activity.

## DISCUSSION

In this study, we demonstrated that overexpressing CXCL10 promotes GBM progression. Furthermore, we showed that calycosin inhibits growth, migration, and invasion in GBM by downregulating the CXCL10 pathway *in vitro*. We also confirmed the anti-tumor effect of calycosin in a GBM xenograft mouse model, aligning with our *in vitro* findings.

The current standard therapy for GBM primarily involves maximal safe surgical resection, supplemented by chemoradiotherapy. However, complete eradication of glioblastoma is challenging due to its multifocal and aggressive invasion [1]. Therefore, the development of new therapeutic agents is urgently needed. In this context, we established that calycosin, a bioactive compound extracted from Radix astragali, exerts its anti-tumor function by downregulating CXCL10 expression in GBM.

Calycosin has been recognized for its anti-tumor effects in various cancers, including glioma [8–10]. Previous studies have shown that calycosin inhibits GBM progression by downregulating TGFβ and c-Met [10, 11]. Additionally, Ni Q et al. reported that combining calycosin with temozolomide enhances anti-glioma effects [24]. However, the precise molecular target of calycosin in GBM remains unclear. Our research reveals that calycosin suppresses GBM growth both *in vitro* and *in vivo* by inhibiting the CXCL10 signaling pathway, suggesting CXCL10 as a potential target for GBM treatment.

CXCL10 plays a crucial role in regulating multiple inflammatory signaling pathways and is intimately linked to the onset and development of various tumors. However, its role in GBM progression is debated. Maru, SV et al. reported that overexpressing CXCL10 significantly influences the proliferation of glioma cells [25]. Additionally, previous studies using immunohistochemical staining have shown CXCL10 overexpression in GBM [26]. Kenji Shono et al. found that CXCL10 downregulation contributes to anti-tumor effects in a malignant glioma mouse model. These findings suggest that CXCL10 is instrumental in GBM growth and progression, and its inhibition may serve as a potential therapeutic target for glioblastoma [27]. Contrarily, other studies have indicated that upregulating CXCL10 could inhibit glioma progression [28, 29]. In our current study, we demonstrated high CXCL10 expression in glioma, negatively correlating with patient prognosis. This supports the notion of CXCL10 as an oncogene in glioma. Furthermore, we identified CXCL10 as a potential target for calycosin, which downregulates the CXCL10-related inflammatory signaling pathway to suppress GBM progression.

Numerous agents show anti-GBM effects *in vitro* but are ineffective in GBM orthotopic xenograft mouse models due to the blood-brain barrier (BBB). Previous research has shown calycosin’s therapeutic effects in cerebral ischemic and brain reperfusion injury models, along with a favorable safety profile [5]. In this study, we found that calycosin inhibited glioblastoma growth by downregulating the CXCL10 signaling pathway in a subcutaneous GBM model, consistent with our *in vitro* findings. Significantly, calycosin markedly reduced tumor volume without affecting body weight, validating its safety and efficacy *in vivo*.

The molecular docking data suggest a potential interaction between calycosin and CXCL10, marked by a strong binding affinity. Such an interaction implies that calycosin could modulate the activity of CXCL10, which may have therapeutic implications. However, these *in silico* findings necessitate further empirical validation. Subsequent experiments, including *in vitro* and *in vivo* studies, are essential to confirm the binding and to understand the impact of calycosin on CXCL10’s biological functions.

## CONCLUSIONS

Overall, we have demonstrated that CXCL10 functions as an oncogene and identified it as a potential therapeutic target for calycosin in glioma treatment.

## MATERIALS AND METHODS

### Reagents

Calycosin was obtained from Tianjin Wanxiang Hengyuan Science and Technology Ltd., Tianjin, China. Dulbecco’s Modified Eagle Medium (DMEM) and Fetal Bovine Serum (FBS) were procured from Gibco™. Antibodies against CXCL10, NLRP3, and IL-1β were sourced from Cell Signaling Technology (MA, USA), while antibodies for NF-κB were acquired from Wuhan Servicebio Technology Co. Ltd., Wuhan, China.

### Patients and sample

Specimens of tumor and adjacent tissues were collected from 24 patients in the Second Affiliated Hospital of Guangzhou Medical University, who had undergone curative surgery from 2015 to 2018 in our hospital, which was approved by the Institutional Ethics Committee in the Second Affiliated Hospital of Guangzhou Medical University. Proteins (24 pairs) were isolated from frozen tumor tissues and adjacent tissues for western blotting assay to assess the expression of CXCL10. Written informed consents were acquired from each patient relying on guidelines of the Declaration of Helsinki. The inclusion and exclusion criteria for collecting glioma samples may include the following:

Inclusion criteria:
Confirmed diagnosis of glioma;
Patient consent to participate in the study;
Not having received radiation, chemotherapy, or surgical treatment, or a certain time interval after treatment.
Exclusion criteria:
Presence of other diseases or history of diseases, such as autoimmune diseases, malignant tumors, etc.;
Use of drugs that may affect the study results, such as steroids, anti-inflammatory drugs, etc.;
Have significant organ diseases such as heart, liver, kidney, etc.;
Pregnant or lactating women.

### Cell culture

Human GBM cell lines U87, U251, and HNA were obtained from iCell Bioscience Inc., Shanghai, China. These lines were cultured in DMEM supplemented with 10% FBS and maintained at 37° C in a humidified 5% CO2 atmosphere.

### Cell viability assay

4000 cells per well were seeded in a 96-well plate and treated with varying calycosin concentrations for 24 hours. Subsequently, 10 μl of CCK-8 solution (Beyotime, Shanghai, China) was added, and incubation continued for 1 hour at 37° C. Absorbance was measured using a Multimode Reader.

### Colony formation assay

500 cells per well were plated in 60mm dishes and treated with different calycosin concentrations for two weeks. Colonies were fixed with methanol and stained using Crystal Violet Staining Solution (Beyotime, Shanghai, China).

### Cell invasion assay

For the cell migration and invasion assay, a transwell system (Corning, NY, USA) was used. 2×105 cells per well in 200 μl DMEM (1% FBS) were seeded in the upper chamber (8 μm Pore Polycarbonate Membrane) coated with 100 μl Matrigel (BD Biosciences, CA, USA). The lower chamber was filled with 600 μl DMEM (20% FBS) and different calycosin concentrations. After 24 hours, cells in the lower chamber were fixed with methanol and stained using Crystal Violet Staining Solution (Beyotime, Shanghai, China). Cells were photographed in five independent fields per well at 100× magnification and counted.

### Reverse transcription polymerase chain reaction (RT-PCR)

Total cellular RNA was extracted using Trizol reagent (Sigma-Aldrich). First-strand cDNA synthesis was performed using a PrimeScripTM RT reagent Kit, followed by PCR with Taq DNA polymerase (Takara, Dalian, China) and specific primers: human CXCL10: 5’-GTGGCATTCAAGGAGTACCTC-3’ (forward) and 5’-GCCTTCGATTCTGGATTCAGACA-3’ (reverse); human GAPDH: 5’-GGAGCGAGATCCCTCCAAAAT-3’ (forward) and 5’-GGCTGTTGTCATACTTCTCATGG-3’ (reverse).

### Western blotting

Western blotting was conducted on glioblastoma cell lysates, clinical normal or tumor tissues, and xenograft glioblastoma tissue homogenates. Protein extraction was performed using PRO-PREP™ Protein Extraction Solution (Cell/Tissue) (iNtRON Biotechnology, Korea), following the manufacturer’s instructions. Equal protein amounts were separated by 10–12% SDS-PAGE and transferred onto polyvinylidene difluoride membranes (Merck, KGaA, Darmstadt, Germany). Membranes were blocked with 5% BSA for 1 hour at room temperature, then incubated with primary antibodies overnight at 4° C. Secondary antibodies conjugated with HRP were applied for 1 hour at room temperature, and signals detected using Immobilon Western HRP Substrate (Merck, KGaA, Darmstadt, Germany).

### Transfection

To overexpress CXCL10, glioblastoma cell lines were infected with a lentiviral vector carrying CXCL10-flag or its control vector (OBiO Technology Corp., Ltd, Shanghai, China). For CXCL10 knockdown, cells were transfected with CXCL10 siRNA or scrambled siRNA (GenePharma, Shanghai, China) using jetPRIME® (Polyplus Transfection, France), according to the manufacturer's protocol. The specific siRNA sequence targeting CXCL10: sense 5ʹ-CCUUAUCUUUUCUGACUCUATT-3ʹ; antisense 3ʹ-UAGAGUCAGAAAGAUAAGGTT-5ʹ.

### U87 xenograft mouse model calycosin treatment

Female BALB/c nude mice were obtained from Guangdong Medical Laboratory Animal Center (Guangdong, China). Mice were aged 6–8 weeks and kept under a standard protocol approved by the Laboratory Animal Center of the Second Affiliated Hospital of Guangzhou Medical University. All procedures performed in studies involving animals were in accord with the ethical standards of the Ethics Committee and the IRB number is 2021-ks-15. Each mouse was injected subcutaneously with cultured U87 cells (5×10^6^ cells/mouse) into the dorsum. The tumor size was measured in two orthogonal directions using calipers, and the tumor volume (mm^3^) was calculated using the equation: 1/2×length×width^2^. When the tumors grew to about 150 mm^3^, the tumor-bearing mice were distributed into two groups (n=5 each) and orally fed with calycosin (10mg/kg/day) or vehicle (equivalent amount of PBS) Tumor sizes and body weights were measured once every 5 days. At the end of these experiments, the mice were sacrificed and the tumors were resected and homogenized for western blotting.

### Molecular docking

Calycosin was designed using ChemBio3D Ultra 14.0, with an initial structure obtained from PubChem based on its CAS number. Energy minimization was performed, and the structure was saved in “mol2” and subsequently “pdbqt” format after processing with AutodockTools-1.5.6. The CXCL10 protein was prepared by removing crystallization water and original ligands using Pymol 2.3.0, followed by charge computation and atom typing with AutoDocktools, and saved in “pdbqt” format. Binding sites were predicted using POCASA 1.1, and molecular docking was executed with AutoDock Vina 1.1.2, where the search space dimensions were set to a grid of 60x60x60 with a grid spacing of 0.375Å and an exhaustiveness of 10. Finally, the docking result was analyzed for interaction mode using PyMOL 2.3.0, allowing for a detailed visualization and understanding of the molecular interactions between calycosin and CXCL10 protein.

### Statistical analysis

Data represent three independent experiments and were analyzed using SPSS 20.0 software. Simple comparisons between two groups were performed using independent T-tests, while multiple group comparisons utilized one-way ANOVA, followed by post hoc Dunnett’s T3 or Tukey’s tests. A p-value < 0.05 was considered statistically significant.

### Availability of data and materials

The datasets used and/or analyzed during the current study are available from the corresponding author on reasonable request.

## Supplementary Material

Supplementary Table 1
