# Supplementary material for: Calycosin (CA) inhibits proliferation, migration and invasion by suppression of CXCL10 signaling pathway in glioma
Source: Aging (Albany NY). 2024 Mar 9;16(5):4191–203. doi: 10.18632/aging.205572 (PMC10968673; doi:10.18632/aging.205572)
Supplement: Supplementary Table 1 [file aging-16-205572-s001.pdf]

SUPPLEMENTARY TABLE

Supplementary Table 1. Clinicopathological characteristics of high and low CXCL10 expression groups.

Characteristics	Low expression of CXCL10 (n=12)	High expression of CXCL10 (n=12)	P-value
WHO Grade			0.0038
Grade II &Grade III	11	3	
Grade IV	1	9	
IDH status			0.0304
Mutant	11	5	
WT	1	7	
1p/19q codeletion			0.0985
non-codel	271	249	
codel	76	95	
Histological type			0.0345
Astroctyoma	2	5	
Glioblastoma	9	1	
Oligodendroglioma	1	6	
Gender			0.6831
Male	7	5	
Female	5	7	
Age			1.0000
<= 60	9	8	
> 60	3	4	
